# Elements of Chemical Philosophy, on the Basis of Reid, with the Requisite Illustrations and Diagrams

**Published:** 1832

**Authors:** 


ART. VI.—*Elements of Chemical Philosophy, on the Basis of Reid, comprising the Rudiments of that Science; and the requisite Illustrations, with Plates and Diagrams.* By THOMAS D. MITCHELL, M. D. Professor of Chemistry and Pharmacy, in the Medical College of Ohio; President of the Ohio Medical Lyceum; Honorary member of the Philadelphia Medical and Columbian Chemical Societies, &c. Corey & Fairbank.

The present volume, almost the first systematic work connected with the profession of Medicine or its associate sciences, which has been published in the west, and emanating as it does from the Medical College of Ohio, seems to demand from the Journal, an extended notice. The liberal endowment which the Medical College has received and continues to receive from the State, demands from the Professors who occupy the exalted honor of a seat in that institution, a corresponding exertion to build up a domestic literature, and inspire a generous emulation in the young physicians of this country to observe, think, and write for themselves, and thus save their country from the degradation of depending upon other countries for the elements of their education. As an indication of a disposition to meet the just expectations of a State which has so liberally patronized them, as an earnest of greater and more elaborate efforts, we have no doubt the present volume will be scanned with an indulgent eye, and will receive a patronage fully in proportion to its merits.

It was our intention to have presented to our readers an elaborate review of the present volume, but having been disappointed in receiving assistance from a quarter in which we were led confidently to believe we should have received it, we are reduced to the necessity of giving it such a common place review as will afford to the public a general idea of its character, without entering into the scientific details which the occasion demands.

*General plan of the work.* The author flatters himself, that he has availed himself of a great improvement upon the general plan of arrangement pursued in chemical works, by adopting that of Dr. Reid, of Edinburgh. At the same time he has supplied some deficiencies in the work of Reid by "borrowing freely from the *course de chimie* of Languier." The advantages of this arrangement are, that commencing with the simple substances, he proceeds to the compound in such succession, as that the history of each is given with all the combinations which it forms, as far as practicable, without at any time anticipating the history of bodies whose properties have not been detailed.

The first chapter, after some general remarks and definitions, is devoted to the consideration of *Attraction, the Laws of Combi-*

*nation, &c.* Chapter II. is devoted to Light and Caloric; of the other imponderable agents, he dispenses with magnetism as altogether foreign to his work, and assigns to electricity and galvanism a separate chapter at the close of the work, as remarkable for its brevity as the space allotted to them in the lectures of his predecessor was for its diffuseness. An arrangement, we think, that by no means comports with their importance as chemical agents.

On the subject of light his ideas are not very accurate nor very perspicuously expressed. For instance, the author says, "the indispensable necessity of light to *perfect* vision is well understood;" we believe we may add, also, the indispensable necessity of light to *imperfect* vision is equally well understood.

Again, he says, "Light cannot be converted into a perceptible mass by any force that we can employ. Its levity is equal to its rapidity, and the one is probably the consequence of the other. Now, how levity can be the consequence of rapidity, or rapidity the consequence of levity, we cannot conceive, having always understood that the *vis inertiæ* of matter induced it to move with a velocity proportionate to the impulse by which it is set in motion, without any regard to its weight.

He appears to favor the opinion, that between light and caloric there is an identity of nature and action, and after detailing a number of instances in which similar results are produced by their agency, he adds, "Here it would seem that caloric and light act precisely alike, and thus far they are identical." It certainly is a very loose mode of reasoning which would infer that all substances which have a tendency to produce similar results must be identical, especially, when the whole amount of the agency appears to be, as it is in the present instance, the removal of an obstacle in the way of chemical action. In the same way might be proved not only the identity of caloric and light, but of caloric and electricity, and of a thousand other chemical agents bearing still less resemblance.

The properties and effects of caloric are illustrated in a very familiar and perspicuous manner. Its radiation and reflection are exemplified by the familiar experiment of two opposite and parallel mirrors. A heated ball being placed in the focus of one, and a thermometer in the focus of the other, the mercury will immediately rise, although the ball by itself would produce no sensible impression even when placed much nearer the thermometer than in the experiment.

"The focal ball does, unquestionably, radiate caloric; for as it is a dull, unpolished body, it cannot reflect. The best reflection viz. polished metals, are the worst radiators; and the best radiators, as all dull, tarnished bodies, are the worst reflectors, or rather, they do not reflect at all. But what becomes of the caloric radiated or sent out, from the focal ball? We answer, it passes or diverges to the circumference of the corresponding mirror, from which it flies off in straight and parallel lines to the circumference of the opposite mirror, from which, by a natural tendency, it converges to the central or focal point, and there falls on the thermometer, ether glass, or candle, producing the effects previously stated.

The experiment with the concave mirrors, may be further varied, to illustrate the reflection of heat. Thus substitute a lump of ice, for the heated iron ball, and very soon, the thermometer in the focus of the other mirror will be affected, and its fluid will fall. Now, say some, this is proof, that cold is a positive agent, and not the mere abstraction of heat. They contend, that the frigorific particles pass from the ice to the thermometer, and act on it, by a positive power. This, however, is a mistake. Remove the ice from the focal point to a place midway between the mirrors, and the thermometer will not be affected; whereas, on the supposition of a positive frigorific agency, the reverse should occur. The true state of the case is this. The ice, being in its proper focus, receives caloric from the thermometer, which is comparatively, the hotter body. The rays, passing from the thermometer to the circumference of the mirror, then go in straight parallel lines, to the circumference of the opposite mirror, and thence converge, to meet in the ice, which occupies the focal point. The fluid of the thermometer sinks, therefore, because it parts with its caloric, to give it to the colder body."

If these experiments prove that caloric exerts a positive influence, the proof is equally decided in favor of the positive agency of cold. In the professor's argument respecting the influence of cold, there is a perfect misrepresentation of the whole question. Those who say that "cold is a positive agent and not the mere abstraction of heat," do not necessarily contend that the frigorific particles pass from the ice to the thermometer, and act upon it by a positive power. On the contrary, they contend for the privilege of supposing, as the professor does in relation to caloric, that it passes to the nearest reflector, and being reflected from it to the other, is concentrated in the focus of the last reflector. The experiment, if true, cannot by any explanation be made to prove any thing but the radiation of cold, and if we admit this property in cold, we cannot deny it a positive agency.

The manner in which this proof positive is explained away, has always appeared to us destitute of every kind of analogical support. What influence can arise from the fact of "the ice being in its proper focus" to make the rays pass from the thermometer to the circumference of the mirror, then go in parallel lines to the circumference of the opposite mirror, and thence converge to meet in the ice which occupies the focal point. We cannot see why the caloric should choose to travel by a circuitous route forty feet instead of travelling to its object through one-fourth of the distance. We are the more disposed to notice the imperfection of this explanation, because it has servilely passed from one copyist to another, without examination, until it has become as generally received as if it were established upon the solid basis of experiment or analogical reasoning.

In speaking of the capacity of bodies for caloric, the author remarks: "two portions of the same body, in the same condition, have precisely the same capacity for caloric, as may be readily shown by mixing a pound of water at 50 deg. with another at 100. The temperature of the mixture will be exactly 75, or the mean heat of the two pounds." Is this true as a general principle?

Professor M. has the following remarks upon the modus operandi of caloric and cold, as medical agents.

"The medical uses of caloric, and also of cold, or the abstraction of heat, claim a passing note; and the main reason for the remarks to be presented on this subject, is, to correct an error in regard to the modus operandi of *cold*. All agree that heat, whether internally or externally applied, in form of vapor, or water, or in any other way, stimulates the system, and increases the action of the heart and arteries. On the simple principle of its repulsive power, its tendency to expand bodies subjected to its influence, one could hardly anticipate any other result from its application to the human body, than that which is constantly witnessed. These things being so, what must, on common sense principles, be the result of an inevitable abstraction of such an agent? Every one who is prepared to look at things just as they are, rather than to examine them through the spectacles of mysticism, will perceive, at once, that the abstraction of heat must give rise to effects of an opposite character to those which follow its direct application. It results, therefore, as a matter of necessity, that *cold* is a sedative; that is, an agent, (if that term be allowed to a mere negative,) which lessens the vital energies, and would ultimately destroy the principle of life, if not properly counteracted.

All admit that alcohol is a stimulus, because it is impossible to resist the daily evidence of our senses on this subject. An individual will keep his system up to the point of full toned excitement, by pursuing a regular course in the use of this poison. But suppose that some one who has power to restrain this lover of the unnatural stimulus, exerts his influence, so as to stop, instantly, the wonted allowance, and to substitute cold water in its place. Here is a case of abstraction of a powerful stimulus, and look at the result. The man's whole frame indicates that a storm is gathering. His energies flag, and he is completely unnerved. Look at the drunkard, who has delayed, for an hour or two, his morning dram, and see if his system cannot furnish evidence, that the want of the accustomed stimulus operates as a sedative. Why, the unhappy creature has about him a temporary palsy; his hand shakes, his tongue falters, and he grasps the glass, as a drowning man would seize a straw. Look at him after he has swallowed the dose, and, in a few minutes, you discover, that the stimulus has had its accustomed effects; and the conviction is irresistible, that while alcohol is an unnatural stimulus to the drunkard, the abstraction is a tremendous sedative. It is precisely so with heat; its action is always to produce positive effects, while cold, operating in its negative character, always produces corresponding results. It is therefore a palpable contradiction, to say that *cold* is a *stimulus*."

Gentlemen are as liable to err when they look at facts through the spectacles of hypothesis or false analogies, as when they examine them through the spectacles of mysticism. The Doctor asserts that heat acts as a stimulant; first, upon universal consent; secondly, because it must be so on the "simple principle of its repulsive power, its tendency to expand bodies subjected to its influence;" the inevitable inference therefore is, that cold—"the simple abstraction of such an agent" "must be sedative." Last of all, to complete the argument, he brings in the analogy of the influence of ardent spirits upon the animal economy.

Let us now divest this argument of the aid it derives from hypothesis and false analogy, and what is the true state of the question? The animal economy is so constituted, that a certain temperature is essential to vitality—not as a stimulus, but as requisite to the healthful condition of the organs of animal life. Apply cold or heat, and a sensation of pain is produced, and as a consequence of the impression upon the nervous system, an increased action of the powers of animal life takes place. If the appellation of stimulant can be applied to either of these agents, it is certainly more appropriate to the former than the latter. A temperature above the ordinary standard is always debilitating to the general system, and is directly and immediately so. It never increases the strength as alcohol does. A diminished temperature, provided it be within a certain limit, is always conducive to the strength and vigor of a constitution that is unimpaired by disease. The increase of the capillary action, indicated by a glow of heat, is as instantaneous as the sensation of cold. A determination to the surface takes place, animal heat is more rapidly developed, the action of the heart is more vigorous, digestion, absorption, and secretion go on more rapidly. The muscular strength and the power to endure fatigue is greatly increased. Now, in all this, there is no "mysticism." It is the universal language of common sense, founded upon the experience of all mankind.

It is true, that upon a subject of this kind, where the practice is so well settled as it is in relation to the remedial effects of cold, hypothesis is of little consequence. So much dissention prevails in the republic of literature, that speculations, little important in themselves, sometimes become serious affairs on account of the language in which they are couched.

*Chapter* III. contains a brief and perspicuous account of the principles upon which the nomenclature of Lavoirsier is founded, with the alterations which subsequent discoveries have rendered necessary.

Professor Mitchell was fortunate enough to advance an opinion twenty years ago, which the progress of chemical science has since amply confirmed, viz. that there is nothing in nature corresponding to Lavoisier's "Acidifying principle."

"Formerly it was supposed, that all acids contained oxygen, as an *acidifying principle;* but as the union of chlorine, iodine, &c. with hydrogen, gives rise to acids, we now distinguish between *oxacids* and *hydracids.* Thus, sulphur, chlorine, iodine, bromine, &c., in union with oxygen, form sulphuric, chloric, iodic, and entirely apart from the influence of oxygen, form hydrosulphuric, hydrochloric, hydriodic, and hydrobromic acids. Whenever the term *hydro* is prefixed to an acid, we are to regard it as an abreviation of hydrogen, which substance is a constituent part of the acid.

On the subject of an acidifying principle, I have given my views at length some years ago. It may not be amiss, however, to state, in this place, that the advances which chemical science is constantly making, have confirmed my early opinions on this point. I repeat, that the term acidifying principle is utterly unphilosophical, not only as applied to oxygen, but to hydrogen, and to every agent which may be supposed to exert an influence in developing acid properties. Every result in nature or produced by art, is a relative effect, and every item concerned, remotely or directly, in the accomplishment of the end, is essential to that end. Hence, I insist, that if an acid be discovered, which shall compose fifty component parts, all of which are requisite in the formation of the compound, the only characteristic of which is acidity, I may affirm with equal propriety of any one, as of any other, of its constituents, that this or that is the acidifying principle. Abstract from the compound either of its parts, and you destroy the peculiar, distinctive character of the acid."

Upon the same ground he rejects the opinion of a principle of alkalinity and of inflammability. It is, indeed, very true, if the opinion of modern chemists is correct, that oxygen is not essential to acidification; and yet the course of reasoning upon which this advance beyond the opinions of the age was predicated, does not appear to have resulted from a very perfect understanding of the use of the term "*principle.*" It never, we presume, entered into the head of Lavoisier to convey the idea that the acids could be formed by oxygen alone; on the contrary, the specific name carbonic, sulphuric, &c. &c. indicates very clearly that Lavoisier meant no more than to point out oxygen as necessarily present in the formation of an acid. And the very slight alterations which are necessary to adapt his nomenclature to the present improved condition of Chemistry, serve to confirm the correctness of the principle upon which that nomenclature was established. Oxygen is indispensably necessary to the formation of almost all acids, and upon this fact was founded Lavoisier's nomenclature. A very few simple substances have since been discovered, which, by an union with hydrogen, form compounds, presenting the characters of acids in a greater or less degree; and these form exceptions to his theory, which still, however, is perfectly adapted to the explanation of almost all the phenomena which the science of Chemistry took cognizance of in his day. The discoveries which have been made since his time consist rather in the collection of a great number of isolated facts, than in the arrrangement of them under any general system of classification. Perhaps we may say of Chemistry, that, as a science, it is confined in its application to the simplest combinations of unorganic matter; and that in proceeding to apply its principles to the complicated combinations of the vegetable and animal kingdom, we find ourselves at every step surrounded by anomalies which we are unable to explain. The following remarks upon the theory of combustion, in a subsequent part of the volume, place the Professor's views in a more favourable aspect.

"Lavoisier probably erred, in accounting for the heat and light evolved in combustion, by reason of the limited view he took of the subject. It would require an infinite number of experiments, to determine with precision the source of light and heat in all cases of combustion, and the precise quantity of these evolved, in each instance, from each body submitted to the process. We say, by way of general remark, that light and heat are properties of all sorts of matter, and that when these are evolved during combustion, it does not arise from any new creation, but from their simple disengagement from the burning bodies, in which they exist by nature, as inherent attributes.

If discovery, in regard to supporters of combustion, had ceased with the production of oxygen, the Lavoisierian system had retained its popularity to the present hour. But the inquiring curiosity of the chemist was not satisfied with present attainments, and his diligent researches soon brought to light, chlorine, iodine, and bromine. In the honor of these discoveries Sir Humphrey Davy had the largest share, and the world is deeply indebted to him for the fruits of his untiring zeal.

The announcement of chlorine and its striking peculiarities, constituted the reasons for what has been designated, *the chloridic theory.*

As *oxymuriatic acid*, this substance had been familiar, as a supporter of combustion; but its powers in that character were ascribed to the presence of oxygen, its compound nature being then universally acknowledged. And such was the tenacity with which long-cherished opinions were held, that the compound quality of oxymuriatic acid was contended for, long after the publication of experiments proving the elementary nature of that body. The most able opponent of the simple character of oxymuriatic acid, was Murray, and for a while he enlisted many on his side of the question. My own inaugural dissertation was, in part, an attempt to disprove the simple character of chlorine, but I am happy to acknowledge, that so far as experiments appear to go, we are bound to regard chlorine as an element.

In this state of things, it ceased to be true, that oxygen was the only simple substance possessing the attribute of supporting combustion. But chlorine did not long stand alone, but was followed by iodine, bromine, sulphuretted hydrogen, and others, which, under given circumstances, evidently maintain the character of supporters of combustion.

These apparent exceptions to the spirit of the Lavoisierian system, seem rather to accord with it, or to be extensions of it. All of them are at war with the notion of the phlogistic system, that combustibles, in burning, become lighter. And even in their bearings on the doctrine of acidification, they present analogies, rather than contrarieties, to the anti-phlogistic system.

In conclusion, we regard combustion as the result of the action of material particles upon each other, as influenced by strong chemical attractions, or opposite electrical conditions, and that it is a necessary consequence of an intense and violent motion between the molecules of matter, whatever be the exciting cause. As light and heat are inherent properties of all matter, we hold, that the evolution of these is not a new product, growing out of the act of combustion, but a mere disengagement or disturbance of these essential constituents of material particles.

In proceeding to the descriptive part of the work, we have few remarks to make. The author's descriptions are generally very perspicuous, his experiments appropriate, and his illustrations peculiarly happy. His method of exemplifying the doctrine of definite proportions, and applying its principles to the analysis and composition of the objects of chemical investigation are very striking, and cannot fail to impress the subject indelibly upon the mind of every attentive reader.

In conformity with the author's general plan of arrangement, the vegetable acids and alkalies occupy a conspicuous situation in the body of the work, following immediately the history of the gases of which they are composed. In conformity with this principle of arrangement ammonia should, perhaps, have occupied the same position. The resemblance, however, of this latter substance to the fixed alkalies, determined the author to place it in connexion with them; an arrangement which derives still further countenance from the experiment of Berzelius, which seems to prove that it, like the other alkalies, has a metallic base.

The account of the vegetable alkalies is very full and complete, but as this subject has been repeatedly referred to in this Journal, we shall not at present allude to it.

Professor M. thinks that injustice has been done to Lavoisier and other Chemists, by ascribing the honor of discovering the metallic base of the alkalies exclusively to Sir Humphrey Davy. Lavoisier suspected it, and Professor Woodhouse, of the University of Pennsylvania, seems to have reduced potash from its oxyde.

"There can scarcely be a doubt, that Professor Woodhouse (formerly of the University of Pennsylvania) was the first person who actually reduced potash to the metallic state. He was operating with pearlash and soot, exposed to a very high degree of heat in a covered crucible, and perceived, that water thrown on the mixture, when quite cold caused it to take fire. He repeated the experiment with charcoal, and the same result followed. He had never seen potassium, nor heard of it; but who will now entertain a doubt, that he actually decomposed potash in the experiment which seemed to him quite unintelligible, and that the combustion which occurred under his notice, was the effect, so often seen in latter times, of the contact of potassium with water."

Honor to whom honor is due. Sir H. Davy certainly has a sufficient basis for his fame, without detracting from the merits of his distinguished predecessors.

As the change in the nomenclature, which has resulted from the discovery of the simple character of chlorine, has produced some confusion in the use of medical terms, we subjoin the following account of Chloride of Sodium, for the purpose of enabling those who are familiar only with the ancient nomenclature, to accommodate themselves to the change of language which the extension of the science has introduced.

"*Chloride of Sodium.*—This is composed of one equivalent of chlorine, 36, and one of sodium, 24. It may be made by burning sodium in chlorine, or by heating it in muriatic acid gas. In the latter case, it effects a decomposition, taking chlorine from the muriatic acid and setting its hydrogen at liberty. If a solution of common salt (muriate of soda) be evaporated, crystals of the chloride are deposited; for this salt, like the muriate of potash, can exist, as such, only in solution, and is converted into a chloride by expelling the water, so as to reduce the whole to crystals. We are, therefore, to regard the solution of common salt as a true muriate of soda, and the same solution reduced by evaporation to the crystallised state, as a chloride of sodium. When water is added to the chloride, that liquid is decomposed; its oxygen joins the sodium to form the protoxyde of soda, while its hydrogen unites to the chlorine to form muriatic acid, and the muriatic acid combines with the protoxyde of soda, to make the muriate of soda. Sodium has a much stronger attraction for chlorine than for oxygen, and hence, soda or its hydrate is decomposed by chlorine, and a chloride of sodium is formed.

The following account of an article, at present much in use as a disinfecting agent, will be acceptable to many of our readers.

"Chloride of sodium is sometimes confounded with *chloride of soda*, but they are different articles. The latter seems to offer a departure from the analogy, generally observed, between the compounds of oxygen, and those of chlorine, iodine, &c. The chloride of soda is, evidently, a chloride of an oxyde. and it is certain, we have no instance of an oxyde of a chloride. For this reason, it was at first doubted, that chlorine could combine, chemically, with either lime or soda, and I do not believe that a true chemical union exists. It is most likely, that the lime and soda retain the chlorine, pretty much as charcoal retains large bulks of various gases, or as water holds in solution ammoniacal gas. Dr. Ure speaks of the *absorption* of chlorine by the hydrate of lime, and it certainly remains to be shown, that any other state of union exists in these chlorides, than that of simple mixture. The evidence in favour of the chemical composition of atmospheric air is, in my view, far stronger than any that has yet been adduced, to prove chloride of soda is a true chemical compound.

Pure chloride of soda is prepared, by passing a current of chlorine gas into a cold and dilute solution of caustic soda, until no more gas can be taken up. Common carbonate of soda may be used in place of the pure alkali; but considerable excess of chlorine must be employed, in order to displace the whole of the carbonic acid."

The author's account of the *Mineral and Vegetable Poisons*—of their tests, antidotes, &c. is very satisfactory. We subjoin the following mode of procuring a solution of arsenic in cases of supposed poisoning, free from admixture with animal and vegetable matter.

"Though considerable information may be obtained, by applying the tests we have described to mixed solutions, suspected to contain the poison, and when they all concur, in the indications which they give of arsenious acid, little doubt can be entertained of its presence; still, in order to avoid every source of fallacy, it will be necessary to continue our investigation still further, separating the matter that appears to have produced the characteristic precipitate with the arsenious acid, and extracting the metal itself, if any arsenious acid shall have been present.

For this purpose, Dr. Christison recommends sulphuretted hydrogen to be employed, dispensing with the other tests; the liquid should be boiled and filtered in the first place, and then acidulated with muriatic or acetic acid, to prevent any alkaline matter, that may be present, from interfering with the precipitation; after which, a stream of sulphuretted hydrogen should be passed through the liquid, and continued, at least, for half an hour; it is then to be boiled, for a few minutes, to expel any excess of sulphuretted hydrogen, and the precipitate collected on a filter, washing it repeatedly with water, and drying it afterwards, by heating it at a temperature of 212 deg. On mixing it intimately with about twice its weight of black flux, and exposing it to heat in a glass tube, over a spirit lamp, the potassium in the black flux combines with the sulphur, and the metallic arsenic is sublimed in the same manner, as in the reduction of arsenious acid, by the same substance. The size of the tube must be adapted to the quantity of the precipitate which has been procured; the most convenient size is about three or four inches long, and about a quarter or a half inch in diameter; the mixture should not fill more than half an inch of the lower part of the tube, and smaller tubes should be used, when only a very minute quantity of matter has been precipitated.

If a crust of metallic arsenic should be obtained, its steel-gray lustre, its brittleness, the facility with which it is volatilised, and the garlic odor that is, at the same time, produced, will be sufficient to distinguish it from any other substance; if, however, there are only very indistinct appearances of the metallic arsenic, the following is the method that I have found most satisfactory, for ascertaining if arsenic be really present. The tube is to be exposed again to heat, over the spirit lamp, till the matter that has been sublimed, is carried a little farther up the tube and completely separated from the black matter that remains at the bottom; the lower part of the tube must then be broken off, drawing a file across it, previously, that it may be easily removed, and the upper part put into another glass tube and boiled, for five or ten minutes, with a little water, to which a few drops of nitric acid have been added. If the tube be coated with any arsenious acid, it will be immediately dissolved, and if any metallic arsenic should be present, it also will be converted into arsenious acid, attracting oxygen from the nitric acid, and being dissolved at the same time, so that the liquid may now be considered as a solution of arsenious acid in water, with a small quantity of nitric acid; and accordingly, on neutralising the excess of acid, by dropping ammonia into it, through a test-tube drawn out at the extremity over a spirit lamp, so as to represent a small funnel terminating in a capillary tube, the ammoniaco-nitrates of copper and silver will produce the characteristic green and yellow precipitates. If they should give no precipitates, then we may conclude, that the mixture which we have examined, contains no arsenic.

Digest a quarter of a grain of metallic arsenic in a test-tube, with a drachm of water and two or three drops of nitric acid, and neutralise the solution with ammonia, in the manner described in the preceding paragraph. Put a number of drops of the solution, on different parts of a sheet of white paper, and touch them with glass rods, dipped in the different solutions used for the detection of arsenious acid, and the characteristic color will appear in each. We must be careful not to dip the same glass rods into different solutions, without previously removing any liquid that may be still adhering to them.

The indications, given by the different tests, are free from every source of fallacy, when they are added to solutions prepared in this manner; as the arsenious acid is thus removed from all the animal and vegetable matter, that might have interfered with their action in the mixed liquid. Infusions of astringent matter and some other vegetable and animal substances, which are frequently met with in the liquid contents of the stomach, have been shown, by Dr. Christison, to be capable of retaining, in solution, the precipitates that arsenious acid gives with the ammoniaco-nitrates of copper and silver. Tartaric and acetic acids appear to be solvents, in some cases, arsenite of silver being soluble in both.

Orfila has proposed to use chlorine, and Mr. Phillips animal charcoal, to decolorise mixed fluids, suspected to contain arsenious acid, and allow the usual tests to be applied in the liquid way, without previously removing the arsenious acid and subjecting it to some process of reduction; but though both may occasionally be used with advantage, it will be better to adopt the method we have already mentioned. It appears, also, from the experiments of Drs. Christison and Paris, that charcoal is capable of precipitating arsenious acid from its solution in water."

We consider it rather a defect in a work designed expressly for medical students, that the chapter devoted to *Animal Chemistry* and the *analysis of Mineral Waters* should be so brief. The same remark may be applied to the section upon Electricity and Galvanism—the whole of which is comprised in the space of four pages—a notice altogether too limited, when we reflect upon their importance as chemical agents.

The present volume we consider to be, in many respects, a very valuable one. The professions of the author are very moderate. His object is simply to prepare for his class and his general readers, a summary of the science, arranged upon a principle, offering, as he conceives, many advantages above the plan generally adopted. It is designed simply as an elementary treatise, and for this purpose we think it has many merits. The style of description and illustration is very good, and as a treatise on chemistry is principally made up of description and illustration, a merit of this kind is the highest recommendation it could possess. In his attempts at reasoning, the author is not always equally happy—his ratiocination being often loose, declamatory, and desultory, and his deductions far-fetched and illogical.

There is another defect in the work, which, although many, and perhaps the most of mankind may think little of it, is a matter of serious consequence. We allude to an occasional habit of speaking in a style of arrogant denunciation towards those who are so unfortunate as to incur the displeasure of the author. The quarrels of authors form no small part of the evils which disturb the peace and harmony of society, and we see no reason therefore, why their conduct towards each other should not be directed by the same rules which regulate the intercourse of men in the ordinary walks of social life.

It would be injustice to the enterprising publishers of this volume, not to notice the neat and beautiful style of execution in which they have presented it to the public. We hope the friends of the American System will liberally patronize this production, so creditable to the Domestic Industry of the West.

